# Impaired Speaking-Induced Suppression Predicts Degraded Agency and Hallucination Severity in Schizophrenia

**DOI:** 10.1016/j.bpsc.2025.10.011

**Published:** 2025-10-28

**Authors:** Songyuan Tan, Yingxin Jia, Miriam Mathew, Namasvi Jariwala, Alvincé Pongos, Kurtis Brent, Judith Ford, Daniel Mathalon, John Houde, Srikantan Nagarajan, Karuna Subramaniam

**Affiliations:** Department of Psychiatry, University of California San Francisco, San Francisco, California (ST, YJ, MM, NJ, JF, DM, KS); Department of Psychology, Palo Alto University, Palo Alto, California (NJ); Department of Otolaryngology, University of California San Francisco, San Francisco, California (AP, KB, JH); Veterans Affairs San Francisco Healthcare System, San Francisco, California (JF, DM); and Department of Radiology and Biomedical Imaging, University of California San Francisco, San Francisco, California (SN)

## Abstract

**BACKGROUND:**

Agency is the awareness of being the originator of one’s own thoughts and actions. Patients with schizophrenia (SZ) show deficits in agency that contribute to distortions in reality monitoring (RM) (distinguishing self-generated from externally produced information) and psychotic symptoms. Agency is also critical for speech monitoring (SM) (monitoring what we hear ourselves say while speaking). For example, disruptions in agency that manifest as hallucinations are thought to result from the misattribution of the source of patients’ inner thoughts/speech as external voices.

**METHODS:**

We used magnetoencephalography (MEG) to assess agency during RM and SM tasks. In healthy control participants (HCs) during SM, the auditory cortical (A1) response is smaller while speaking (speak condition) compared with listening to the same speech (listen condition). This is known as the speaking-induced suppression (SIS) M100 response, which is measured using MEG 100 ms after speech onset.

**RESULTS:**

During RM, patients with SZ (*N* = 30) showed impairments in both self-agency (identification of self-generated information) and external agency (identification of externally produced information) compared with HCs (*N* = 30). During SM, patients with SZ failed to enhance M100 responses during the listen condition, resulting in weakened SIS—that is, smaller M100 listen minus speak differences. Weakened SIS predicted worsening hallucination severity.

**CONCLUSIONS:**

Patients with SZ showed degraded neural M100 responses in A1 during the listen condition, which drove impaired SIS (i.e., smaller M100 listen minus speak differences). Impaired SIS indicated degraded auditory sensory predictions, making it more likely for patients with SZ to misattribute the source of inner thoughts/speech as externally derived, giving rise to disruptions in agency during RM and more severe hallucinations.

Schizophrenia (SZ) is a serious neuropsychiatric disorder affecting ~1% of the population. Patients with SZ show cardinal deficits in agency—the awareness of being the cause of one’s own thoughts and actions (1). Impairments in agency contribute to distortions in reality monitoring (RM) (i.e., impairments in distinguishing self-generated from externally produced events) and result in psychotic symptoms of hallucinations (2–7). Agency is also critical for speech monitoring (SM) (monitoring what we hear ourselves say while speaking). For example, disruptions in agency that manifest as hallucinations are thought to occur when patients misattribute the source of their inner thoughts/speech to external voices (2,3,5,8,9). Antipsychotic medications are far from adequate, with up to 40% of patients with SZ continuing to remain symptomatic (10). For example, although clozapine is considered the most efficacious antipsychotic medication in refractory patients, 40% to 70% of these patients achieve only partial response to it while also experiencing the side effects of antipsychotic medication (11). Thus, there is an urgent need to understand the neural impairments underlying agency deficits that drive psychotic experiences in SZ.

In the current study, we used magnetoencephalography (MEG) to assess agency during RM and SM tasks. Our previous studies revealed that healthy control participants (HCs) showed increased activity in the medial prefrontal cortex (mPFC) during the successful encoding and retrieval of self-generated information on our RM task, which correlated with accurate judgments of self-agency, indicating that mPFC represents one neural correlate of self-agency (5,12). In other words, the more HCs activated the mPFC during encoding of self-generated information, the more accurate they were at subsequent retrieval of this self-generated information. In contrast, while performing the RM task in our functional magnetic resonance imaging (fMRI) studies, patients with SZ showed hypoactivation in the mPFC and left posterior superior temporal gyrus/sulcus (L.pSTG/S) associated with impairments in both self-agency judgments (i.e., during identification of self-generated information) and external-agency judgments (i.e., identification of externally produced information) compared with HCs (4,5,13). In the current study, we examined whether we can extend these fMRI findings using MEG in a different sample of HCs and patients with SZ, and for the first time, we examined whether there is a common mechanism that underlies impairments in SM and RM tasks that results in psychotic symptoms in SZ.

In HCs, normally during SM, the primary auditory cortical (A1) response in the L.pSTG/S is smaller while speaking (speak condition) compared with listening to the same speech (listen condition). This is known as the speaking-induced suppression (SIS) M100 response, which can be measured using MEG at approximately 100 ms after speech onset (2,14–16). In other words, in HCs, self-generated (and thus highly predictable) sounds give rise to suppressed responses (SIS), enabling distinction between self-generated versus external sounds (2,14–16). Therefore, SIS is indicative of highly predictable self-generated forward models (also known as efference copies/corollary discharge neural mechanisms) that allow all animal species to discount sensations resulting from their own actions (2,17,18). In other words, SIS is the phenomena wherein in HCs, self-generated speech is highly predictable and therefore elicits a suppressed response in the left A1 compared with external speech (2,14,16), indicative of a biological basis for self-agency that is essential for normal interactions with outside reality (2,17,18). In contrast, previous studies have shown that patients with SZ reveal impaired M100 A1 SIS responses (i.e., impaired M100 while speaking compared with listening to the same speech) (3,15,16). Here, we examined the mechanisms that drive such a weakened M100 SIS response in SZ, and for the first time, we examined how impairments in low-level M100 SIS responses during SM relate to higher-level agency judgments during the RM task and are linked to psychotic symptoms of hallucinations in SZ. Previous studies have independently shown SIS abnormalities and RM impairments in patients with SZ. In contrast, our novel contribution lies in integrating distinct RM and SM tasks within a predictive coding/efference copy framework to provide a common unitary basis for impairments in agency across distinct low-level SM and high-level RM tasks in SZ, which contribute to psychotic symptoms of hallucinations.

Both self-agency and external-agency impairments in SZ may contribute to hallucination severity. Hallucinations can arise from self-agency deficits, where internally generated thoughts or inner speech are misattributed to external voices, and from external-agency deficits, where patients fail to appropriately enhance auditory responses to external input. In such cases, degenerated efference copy mechanisms to predictable self-generated sounds and impaired auditory responses to less-predictable external sounds would produce similar M100 responses in patients with SZ during listening and speaking. These failures indicate impaired auditory predictions, elevate prediction error, and undermine the capacity to distinguish the source of inner self-generated thoughts/speech from external auditory stimuli, thereby contributing to hallucinations (2,3,5,8,9,16,19,20). Therefore, we had 3 specific hypotheses. First, during the RM task, patients with SZ would show impairments in both self-agency judgments (i.e., impaired identification of self-generated information) and external-agency judgments (i.e., impaired identification of externally produced information) compared with HCs. Second, we hypothesized that during the SM task, patients with SZ would show SIS impairments revealed by a weakened M100 response during the listen condition compared with the speak condition (i.e., impaired M100 listen response minus M100 response during the speak condition). Third, we hypothesized that SIS impairments in patients with SZ would predict agency impairments during RM and worsening hallucination severity. If these hypotheses are confirmed, the current study would provide the first evidence for impairments in agency during a high-level cognitive judgment-based RM task that are linked to M100 primary sensory impairments during a low-level SM task, establishing a unitary basis for the underlying neural mechanisms that contribute to the psychotic symptoms of hallucinations in SZ.

## METHODS AND MATERIALS

### Participants

The current study constitutes the baseline MEG portion of a National Institute of Mental Health–funded study of SZ. Participants were recruited through our ClinicalTrials.gov site (NCT04807530) or from our previous research studies. Patients with SZ (*n* = 30) and HCs (*n* = 30) were matched at the group level on age and gender, and they provided informed consent for this institutional review board–approved protocol. Participants then completed this MEG study and all clinical assessments at the University of California San Francisco (Table 1). Inclusion criteria for patients with SZ were an Axis I SZ diagnosis, assessed with the Structured Clinical Interview for DSM-5; for HCs, inclusion criteria were no psychiatric disorder, no substance abuse, met MRI criteria, age between 18 and 64 years, and English as the first language. Two HCs and 2 patients with SZ were either ineligible or unavailable to complete the MEG portion of the study. Hallucination severity was measured using the Scale for the Assessment of Positive Symptoms (SAPS) (21).

### RM Task

**Figure 1.**
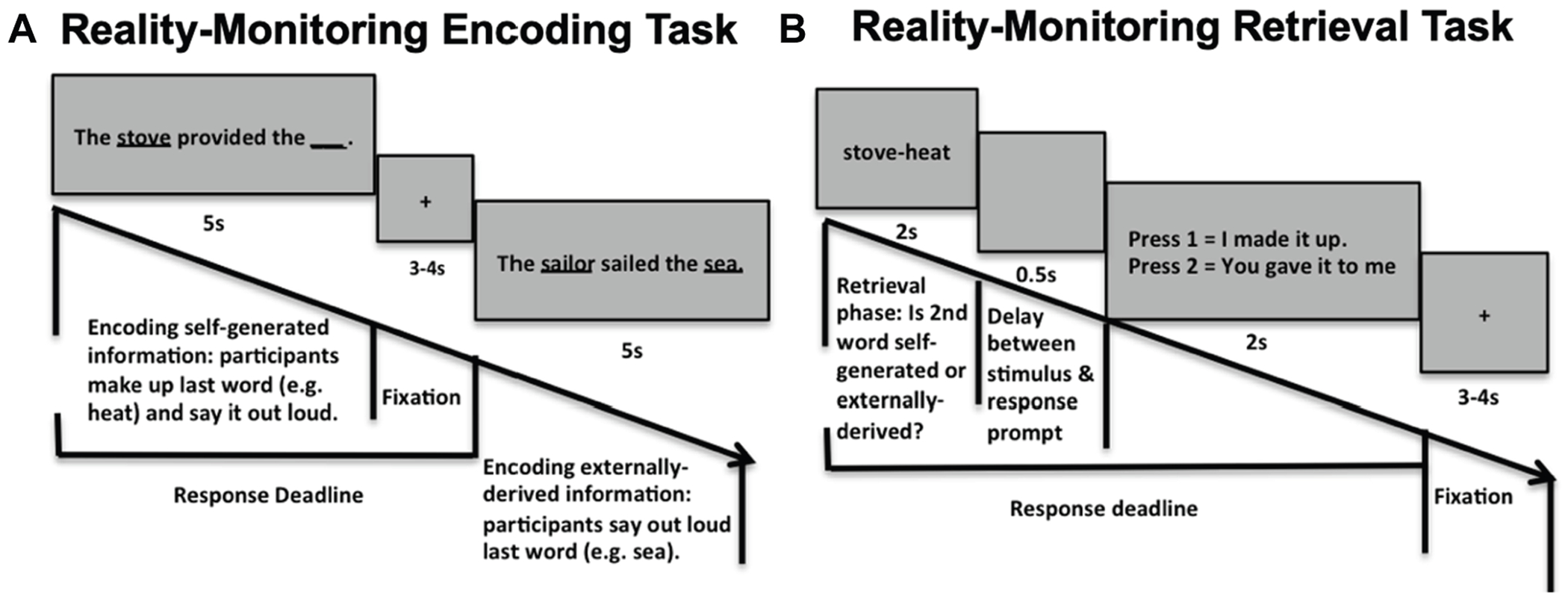
Reality-monitoring task. **(A)** Encoding: Participants were given semantically constrained sentences where on one half of the sentences, the final word was left blank for patients with schizophrenia to generate themselves (e.g., The stove provided the _____), and on the remaining half, the final word was externally derived because it was provided by the experimenter (e.g., The sailor sailed the sea). Participants vocalized the final word of each sentence. **(B)** Retrieval: Participants were randomly presented with the noun word pairs from the sentences and needed to identify with a button press whether the second word was previously self-generated (e.g., stove-heat) (i.e., make self-agency judgments) or externally derived (e.g., sailor-sea) (i.e., make external-agency judgments). We assessed neural activity underlying agency by comparing magnetoencephalography activity during encoding and retrieval of self-generated information with externally derived information.

Participants in the study performed RM and SM tasks in the MEG scanner. As described previously (4,5,22,23), the RM task consisted of an encoding phase and a memory retrieval phase (Figure 1A, B). During the encoding phase, participants were visually presented with semantically constrained sentences with noun-verb-noun format. On half the sentences, the final word was either left blank for participants to generate themselves (e.g., The stove provided the ______) or was externally generated by the experimenter (e.g., The sailor sailed the sea) (Figure 1A). For each sentence, participants were told to vocalize the final word of each sentence, which included both self-generated and externally generated words. The duration of the RM encoding phase was about hour, and the time delay between the end of the encoding phase and the start of the memory retrieval phase was about 5 minutes during which the experimenter incorporated the words generated by the participant to the stimuli for the retrieval phase of the RM experiment. The duration of the retrieval phase was about 30 minutes during which participants were randomly visually presented with the underlined noun pairs from the sentences (e.g., stove-heat) and were asked to identify by pressing a button on the button box whether the second word was previously self-generated or externally generated (Figure 1B). Self-agency judgments were computed as the number of correctly identified self-generated trials, and external-agency judgments were computed as the number of correctly identified externally derived trials.

### SM Task

**Figure 2.**
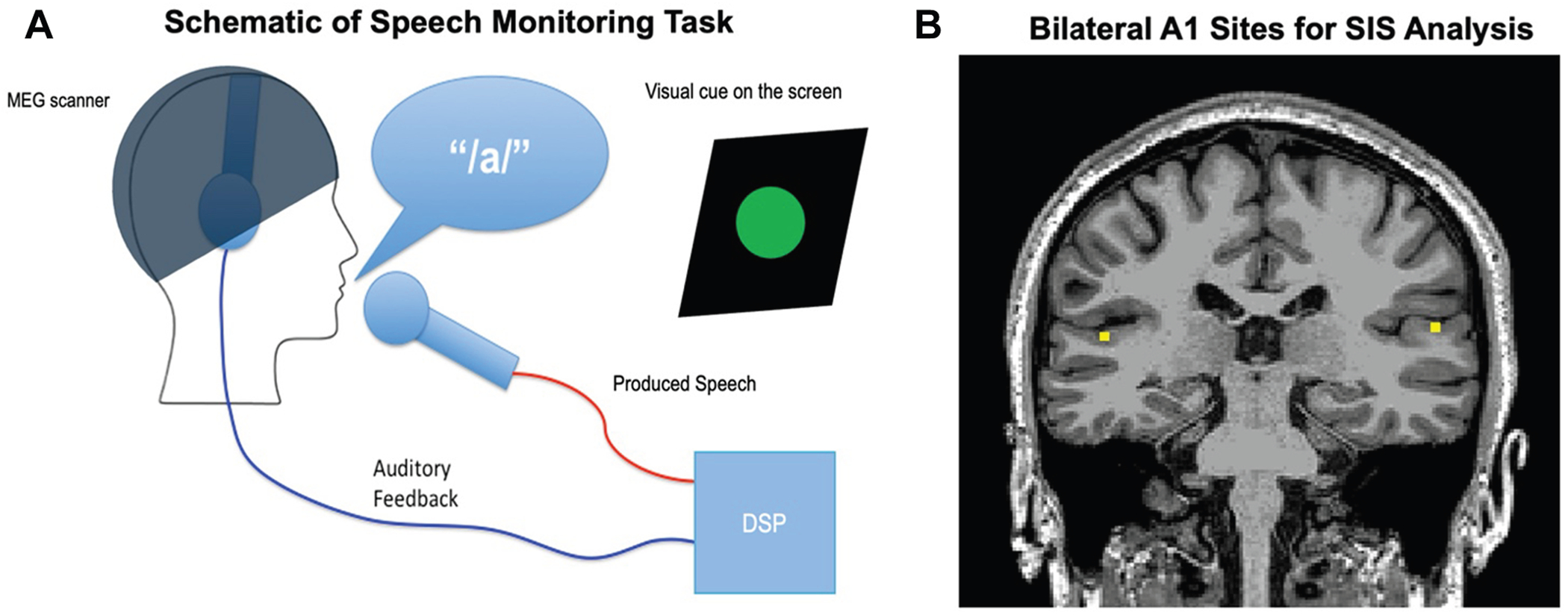
**(A)** During the magnetoencephalography (MEG) speak session, participants vocalized the vowel /α/ when they saw a green dot on the screen, while listening to real-time auditory feedback in headphones via the digital signal processor (DSP). During the MEG listen session, when the green dot appeared on the screen, participants listened to their recorded phonation of the vowel /α/ from the speak session. **(B)** We used MEG to record the M100 response based on magnetic resonance imaging coordinates of the primary auditory cortex (A1) in each participant [see yellow dots in **(B)**] (left hemisphere Montreal Neurological Institute [MNI]: x, y, z = −54, −27, 12; right hemisphere MNI: x, y, z = 54, −27, 12). M100 responses were smaller during self-generated speech (i.e., saying the vowel /α/) compared with when hearing this same speech in subsequent playback. This is known as the speaking-induced suppression (SIS) response.

During the MEG SM task, participants wore an MEG-compatible microphone and a pair of headphones. The microphone was connected to an amplifier, and the amplified audio signal was transmitted back to participants through headphones. Before the experiment, participants confirmed that they could clearly hear the audio signal (Figure 2).

Participants completed 2 sessions, speak and listen. Each trial began with a green dot that appeared on the screen. For the speak session, participants were instructed to vocalize the vowel /ɑ/ when they saw the green dot. They continued the phonation for 2.5 seconds until the dot disappeared while listening to real-time auditory feedback from the headphones. In the listen session, participants passively listened to their own recorded phonation playback from the speak session when they saw the green dot on the screen (Figure 2). Thus, in both speak and listen conditions, participants produced or heard the same sustained vowel sound, (/ɑ/), ensuring that acoustic properties and vowel duration were matched across conditions and between groups. To control for the effects of attention on M100 amplitude, we focused on SIS, calculated as the difference between the M100 response in the listen versus speak conditions (i.e., M100 listen – M100 speak). This subtraction approach minimizes the influence of general attentional differences between conditions. The M100 response was measured using MEG at approximately 100 ms postspeech onset, a time window that is well characterized in previous SIS research and is less susceptible to variability in attention-related cortical activity in top-down attentional modulation than later components, and our subtraction approach (listen – speak) further minimizes attentional confounds.

### MRI Acquisition

Structural T1-weighted MR images were obtained for each participant using a 3T Siemens MRI scanner for MEG source space reconstruction. For each participant, the outline of the brain on the structural scans was extracted, and the segmented brain was treated as a volume conductor model for the source reconstruction described below.

### MEG Data Acquisition

Magnetic fields were recorded using a whole-head 275-axial gradiometer MEG system in a shielded room. Three fiducial coils were placed to localize the head position relative to the sensors. Three fiducial coils were placed to localize the position of the head relative to the sensor array for coregistration of the MEG data with each individual’s structural anatomical MRI. Noisy sensors and trials with artifacts (i.e., due to head movement) were defined as magnetic flux exceeding 2.5 pT. Epochs were rejected from further analysis if they contained artifacts.

### MEG Analyses

An analysis interval of 600 ms was extracted for each trial, which spanned from −300 to 300 ms relative to the phonation onset. For each participant, the M100 response was averaged across all trials for each condition and each hemisphere. Coregistration of the MEG data with each individual’s anatomical MRI was performed based on the 3 fiducial coil positions utilizing NUTMEG. Bayesian covariance beamforming was applied to the average M100 response across all trials for left and right sensor arrays, focused on the left (Montreal Neurological Institute [MNI] x, y, z = −54.3, 226.5, 11.6) and right (MNI x, y, z = 54.4, −26.7, 11.7) A1 cortices in each participant. Source time series was converted into absolute Bayesian covariance beamformer activity (24) at each time point from −100 to 300 ms for a total duration of 400 ms in the left and right A1 using MNI participant-specific coordinates. The extracted time course for each condition (speak and listen) in each hemisphere were normalized as *z* scores for each participant. M100 responses were computed 100 ms after speech onset (i.e., 50 ms before and after the 100-ms time point after speech onset) during the listen condition and the corresponding speaking condition (25). To compute SIS, M100 responses were subtracted for listen compared with speak (i.e., M100 listen minus M100 speak response) at each time point during this 100-ms time window for each participant in each hemisphere.

### Statistical Analyses

One-way analyses of variance (ANOVAs) were implemented to examine group differences between HCs and patients with SZ in M100 responses during SM as well as in self-agency judgments (i.e., self-generated identification accuracy) and external-agency judgments (i.e., externally generated identification accuracy). All ANOVAs included multiple comparison false discovery rate (FDR) corrections (*p*_FDR_ < .05) to control for type I error. Pearson’s correlation tests were used to measure the strength of the linear relationship between M100 responses with agency judgments and hallucination severity. No other brain regions or symptom domains were tested. To control for experiment-wise error, we applied Benjamini-Hochberg FDR multiple comparison correction across 2 prespecified regions of interest (ROIs) in the left and right pSTG/S. Given the modest sample size for ROI-symptom correlation analyses (*n* = 28), we used a *q* < .10 multiple comparison threshold, which is commonly implemented in small-sample exploratory neuroimaging and mechanistic studies to balance type I and II errors (26–28). While this approach reduces the risk of type II error, results should be interpreted with appropriate caution and validated in future studies with larger samples.

## RESULTS

**Figure 3.**
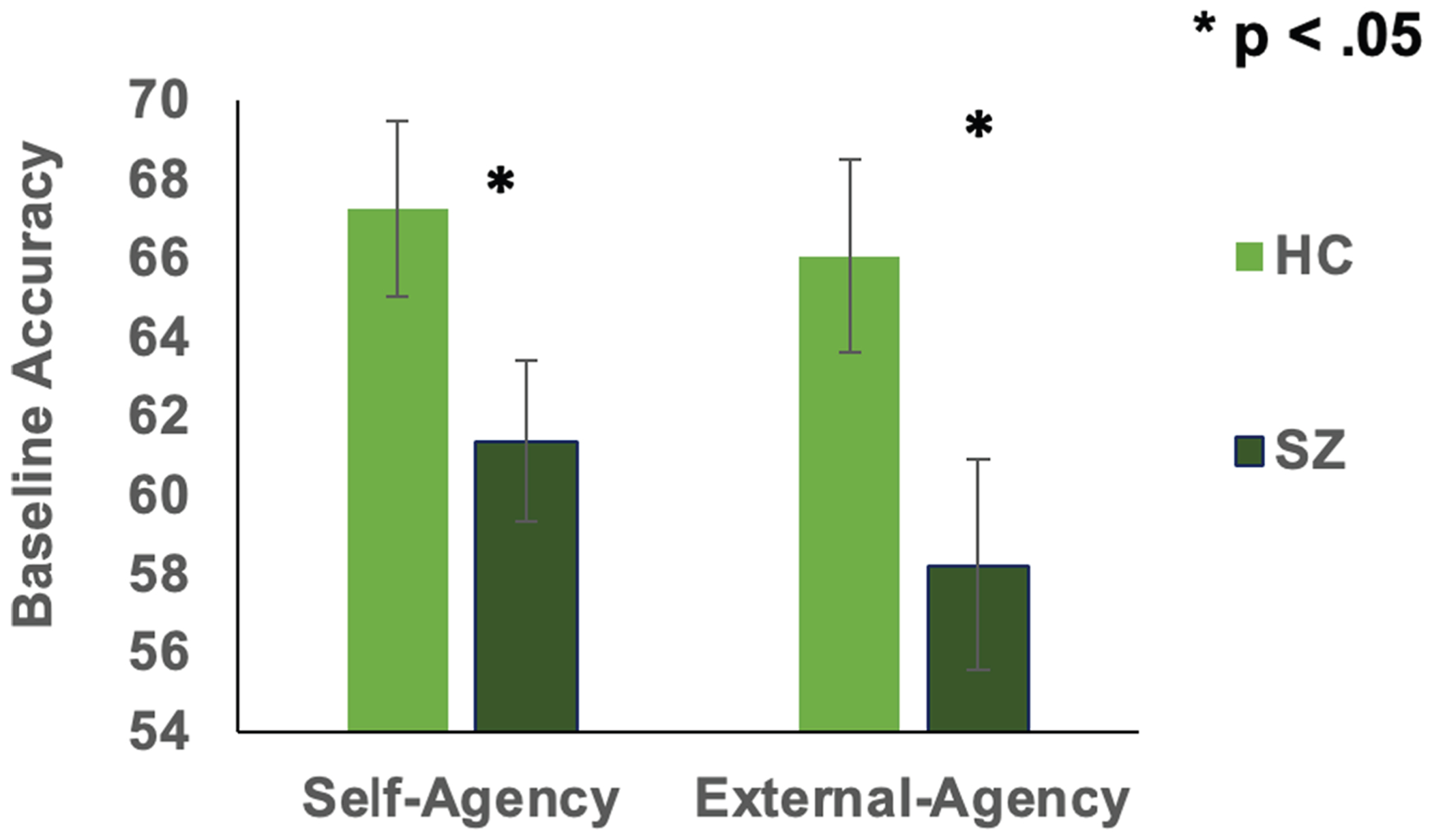
Patients with schizophrenia (SZ) showed impairments in agency during performance on the reality-monitoring task compared with healthy control participants (HCs).

**Figure 4.**
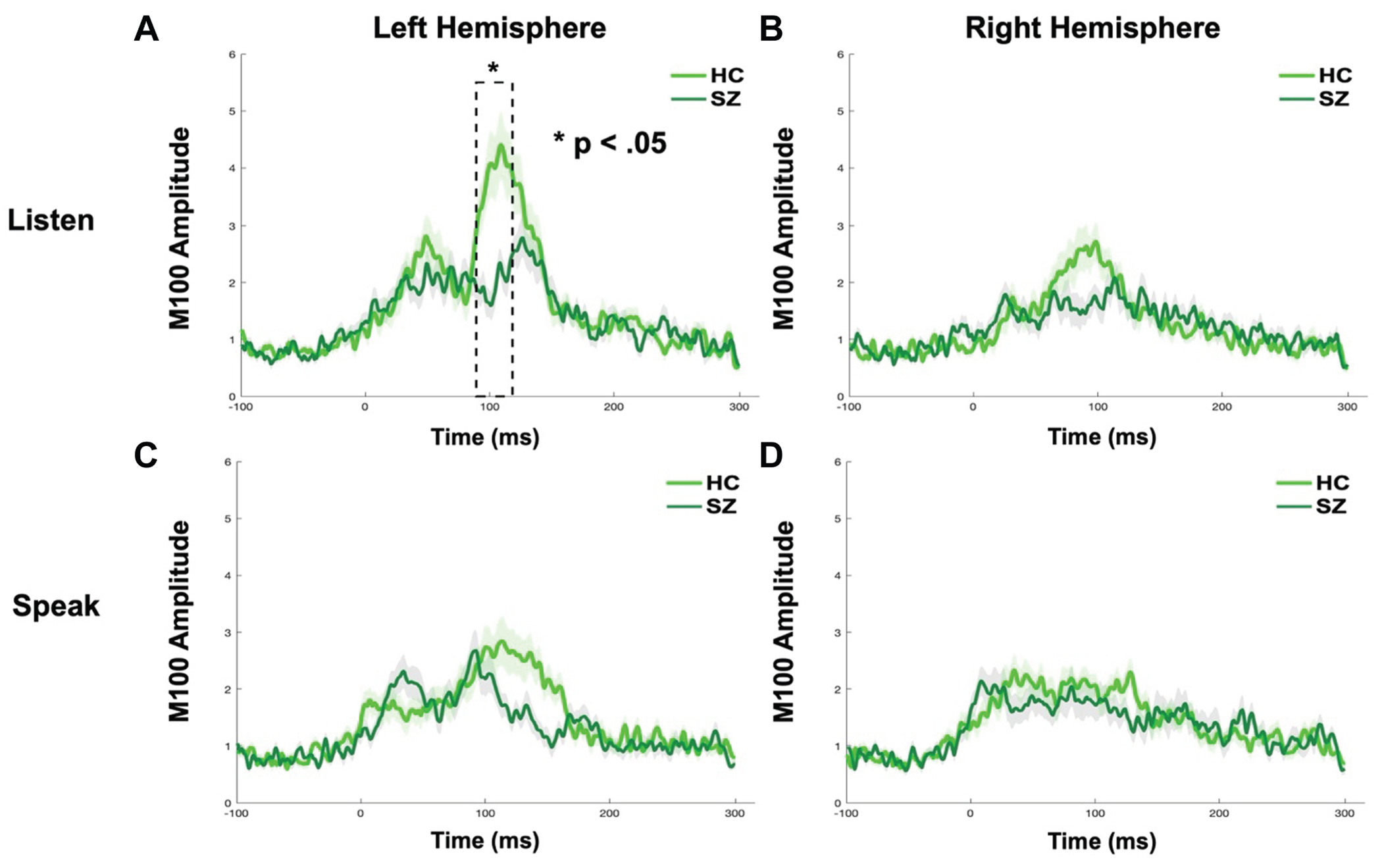
**(A–D)** Compared with healthy control participants (HCs), patients with schizophrenia (SZ) failed to enhance neural M100 responses in the left primary auditory cortex only during the magnetoencephalography listen condition.

**Figure 5.**
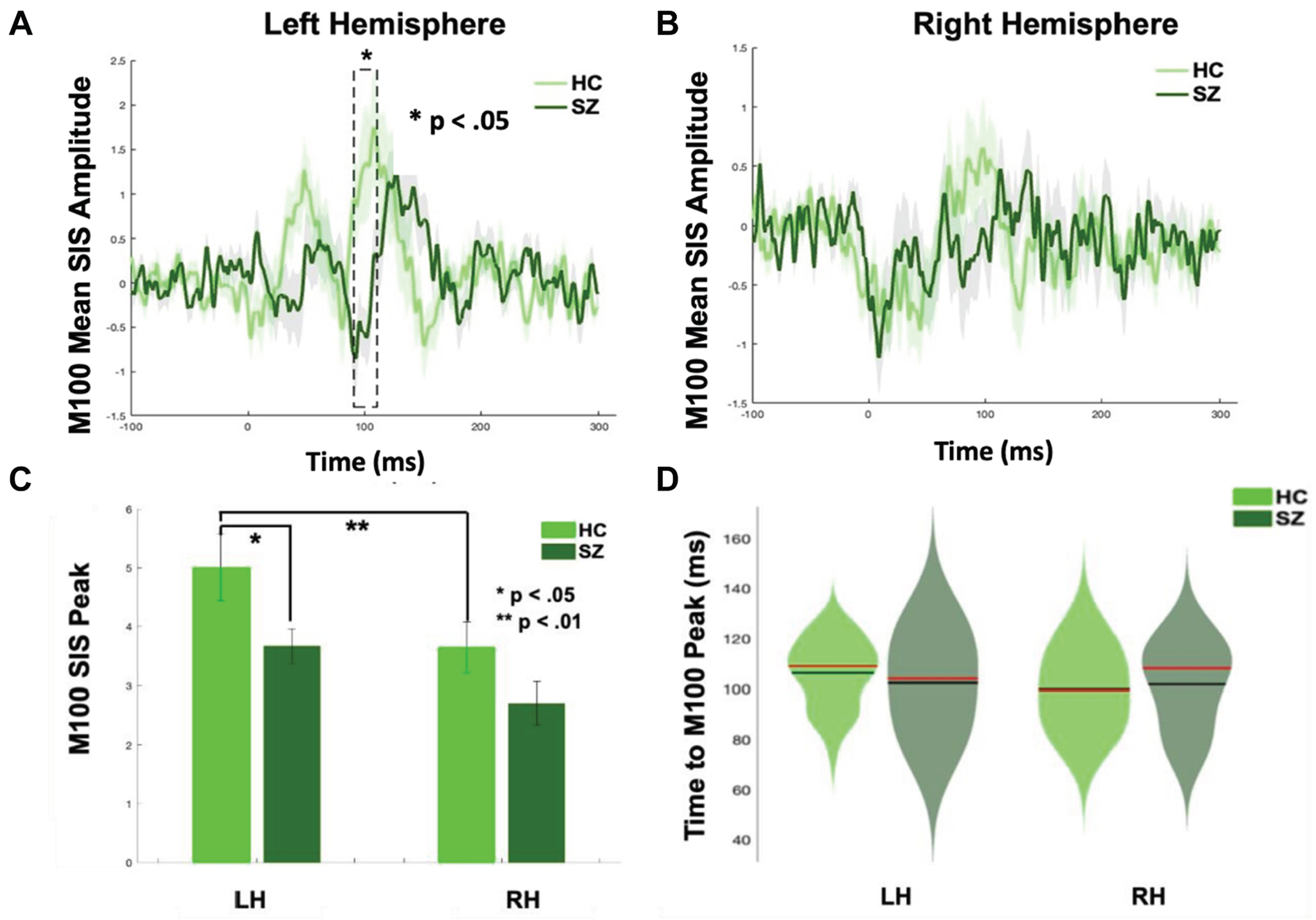
**(A, B)** Speaking-induced suppression (SIS) mean amplitudes (*z* scores) in the left hemisphere (LH) and the right hemisphere (RH) were calculated by subtracting the M100 auditory evoked response during the speak condition at each time point from the listen amplitude (i.e., SIS amplitude = listen – speak). The black dashed box indicates the time window that showed a significant SIS difference approximately 100 ms after speech onset (i.e., 90–110 seconds) only in the LH between the healthy control participant (HC) and schizophrenia (SZ) groups (*p* < .05). Zero ms indicates voice feedback onset in speak and listen sessions. **(C)** Bar charts illustrate a laterality effect in which HCs reveal a significantly larger M100 peak SIS amplitude (i.e., peak listen minus speak *z* scores) in the LH compared with the RH (**p < .01). This laterality effect is absent in the SZ group. HCs also show a significantly greater M100 SIS response in the LH than participants with SZ (*p < .05). **(D)** The violin chart shows that there was no between-group difference or laterality effects in time to reach the M100 peak (median = red line; mean = black line).

Between-group 1-way ANOVAs indicated that on the RM task, patients with SZ revealed significant impairments in both self-agency judgments (*F* = 4.99, *p* = .03) and external-agency judgments (*F* = 7.38, *p* = .01) compared with HCs (Figure 3). Both effects remained significant after controlling for multiple comparisons using the Benjamini-Hochberg FDR correction, with *q* values of .03 and .02, respectively. The effect size of the difference in self-agency and external-agency judgments between HC and SZ was 0.62 and 0.63, respectively. During the SM task in the listen session when participants were passively listening to their recorded phonation from the speak session, between-group 1-way ANOVAs revealed that M00 A1 activation in L.pSTG/S in the SZ group was significantly lower than HC around 100 ms after playback onset (*F* = 5.37, *p* = .024, *q* = .048) (Figure 4A). The effect size of the difference in M100 responses between HC and SZ in the listen session was 0.62. Specifically, SZ showed reduced left M100 responses in the time range from 90 to 110 ms. As shown in previous MEG and electroencephalography (EEG) studies (2,14–16,29), the M100 response in MEG or N1 response in EEG during the listen condition is the standard that is compared with the same responses in the speaking condition (i.e., listen minus speak) to compute the amount of suppression (i.e., the SIS response). Additionally, the reduced M100 response in the listen condition in patients with SZ drove a significantly reduced M100 SIS (i.e., listen minus speak) response (*F* = 10.14, *p* = .002, *q* = .008) within the same 90- to 110-ms time window (Figure 5A). Both M100 listen and SIS M100 effects remained statistically significant in the left hemisphere after Benjamini-Hochberg FDR correction for 4 comparisons across both hemispheres at *q* < .05. The effect size of the difference in M100 SIS responses between HCs and patients with SZ in the listen session was 0.87. In both HC and SZ groups, the M100 listen response was strongly correlated with the M100 SIS response (HC: *r* = −0.98, *p* < .00001; SZ: *r* = −0.93, *p* < .00001). We did not find any between-group differences in M100 response in the right hemisphere or during the speak condition (all *p*s > .80).

We also found that the HC group revealed a laterality effect, with significantly greater M100 A1 activation in L.pSTG/S during the listen condition in the left hemisphere compared with the right hemisphere (*t* = 2.92, *p* = .007, *q* = .01). Greater laterality M100 A1 responses in HCs during the listen condition in the left hemisphere drove a greater M100 SIS (i.e., listen minus speak) laterality response in the L.pSTG/S compared with the right pSTG/S (*t* = 2.81, *p* = .009, *q* = .09) (Figure 5C). After Benjamini-Hochberg FDR correction, both M100 A1 during listen and the SIS laterality effect remained significant at *q* < .05. This laterality effect was not observed in the speak condition in HCs or in patients with SZ in any condition (i.e., listen, speak, or SIS conditions) (all *p*s > .05). We did not find any laterality effects in the time to reach peak M100 A1 responses in either group or any between-group differences in the time to reach peak M100 response (all *p*s > .05) (Figure 5D).

**Figure 6.**
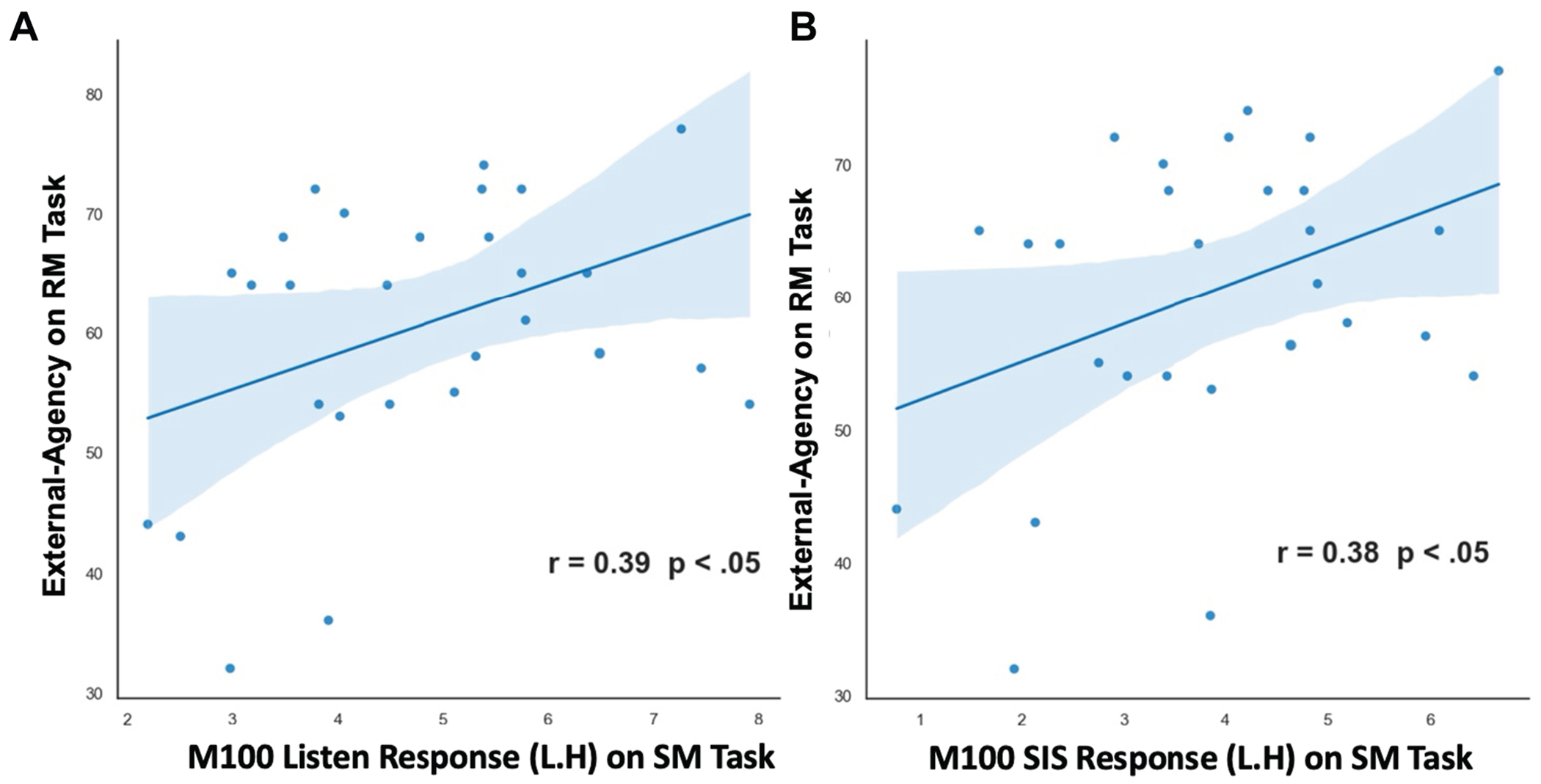
The scatter plots illustrate significant positive correlations between **(A)** magnetoencephalography M100 listen and **(B)** M100 speaking-induced suppression (SIS) (i.e., listen minus speak) responses in the left hemisphere (L.H) during the speech-monitoring (SM) task with external agency during the reality-monitoring (RM) task. The shaded area indicates 95% CIs.

**Figure 7.**
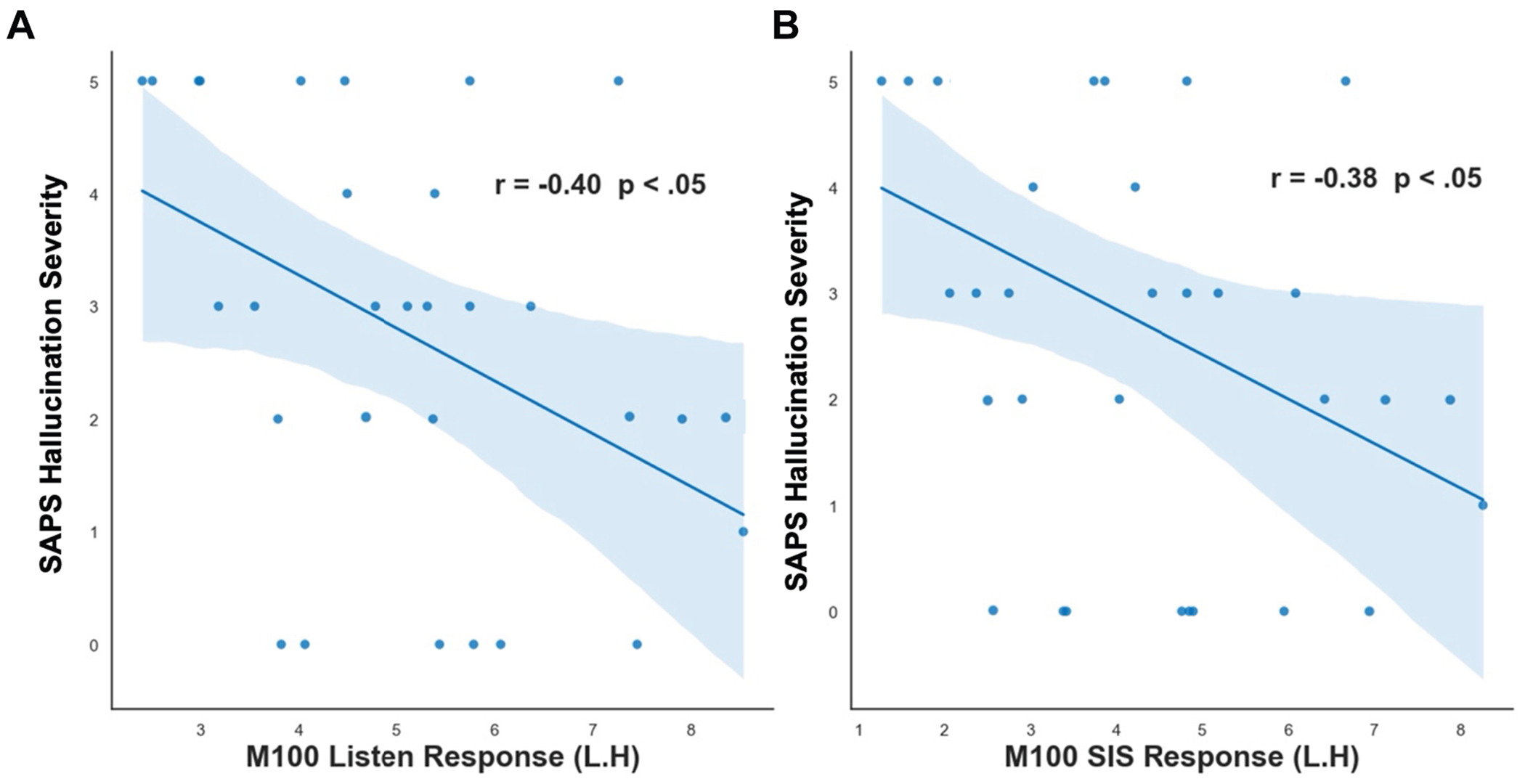
The scatter plots illustrate significant negative correlations between **(A)** magnetoencephalography M100 listen and **(B)** M100 speaking-induced suppression (SIS) (i.e., listen minus speak) responses in the left hemisphere (L.H) during the speech-monitoring task with hallucination severity on the Scale for the Assessment of Positive Symptoms (SAPS). The shaded area indicates 95% CIs.

Interestingly, we found that patients with SZ who showed greater M100 responses in the L.pSTG/S during the listen condition while performing the SM task also revealed greater external-agency judgments during the RM task (*r* = 0.39, *p* = .04, *q* = .08) (Figure 6A). This significant relationship was also sustained for patients with SZ who showed greater M100 SIS responses (i.e., listen minus speak) in the L.pSTG/S, which also predicted greater external-agency judgments during the RM task (*r* = 0.38, *p* < .05, *q* = .049) (Figure 6B). Finally, we found that patients with SZ who showed greater M100 A1 responses in the L.pSTG/S during the listen condition and during SIS revealed lower hallucination severity on SAPS (*r* = −0.40, *p* = .03, *q* = .06 and *r* = 20.38, *p* = .046, *q* = .046, respectively) (Figure 7). All ROI-symptom associations survived Benjamini-Hochberg FDR correction at a threshold of *q* < .10, consistent with thresholds used in small-sample exploratory neuroimaging studies. We did not find any correlations between M100 responses during the listen condition or SIS with agency or hallucinations in the right hemisphere (all *p*s > .05).

## DISCUSSION

In this study, we found that 1) patients with SZ had impairments in both self-agency (i.e., identifying themselves as the originator of self-generated information) and external agency (i.e., identifying externally derived information) on the RM task; 2) patients with SZ had degraded M100 A1 responses in the L.pSTG/S during the listen condition, which contributed to impaired M100 A1 SIS responses; 3) patients with SZ who had greater M100 responses in the L.pSTG/S during the listen condition and greater SIS also showed greater externalagency judgments; and 4) patients with SZ who had greater M100 responses in the L.pSTG/S during the listen condition and greater SIS also had lower hallucination severity. Together, these convergent findings across distinct tasks reveal that the failure to enhance auditory sensory responses to information in the external environment on a low-level SM task suggests that patients with SZ have degraded auditory sensory predictions that impaired their ability to identify externally produced information on a high-level RM task, making them more likely to misattribute the source of self-generated information (i.e., inner thoughts/speech) as being externally produced, giving rise to disruptions in agency judgments and hallucinations (2,3,8,9,15,30). This is the first proof-of-concept study to delineate the neural underpinnings of hallucination severity in SZ by relating auditory sensory impairments in patients with SZ on a low-level SM task that predicted agency impairments on a completely different high-level RM task, which contributed to worsening psychotic hallucination severity.

Our findings in HCs are consistent with previous research revealing that HCs showed stronger laterality M100 responses in the left hemisphere during the listen condition and greater SIS, consistent with left hemisphere dominance for language processing (29,31–35). This stronger laterality M100 response in the left hemisphere was not observed in the SZ group. We did not find any between-group differences in the time to reach peak M100 responses; we found differences only in M100 amplitude responses during the listen condition, but not in the speak condition, that were greater in HCs compared with patients with SZ, which were specific to the left hemisphere. Normally in HCs, M100 responses while speaking give rise to suppressed responses (SIS), thus allowing speakers to enhance M100 responses while listening to sounds in the external world (2,14–16). Therefore, SIS is indicative of highly predictable self-generated forward models (i.e., efference copies/corollary discharge neural mechanisms) that allow all species across the animal kingdom to discount sensations resulting from their own actions (2,17,18). Thus, SIS provides a primordial basis for self-agency that is necessary for normal interactions with the outside world (2,17,18). Here, we found that HCs showed enhanced M100 auditory sensory responses to information in the external environment during the listen condition that drove greater M100 differences between the listen and speak conditions (i.e., greater SIS response). These findings reveal that intact efference copy mechanisms from frontal neural sites to the auditory cortex in HCs enabled the auditory cortex to generate reliable self-predictions about what they expected to hear while speaking, leading to a suppressed M100 response in the speak condition, compared with the listen condition.

In contrast, in patients with SZ, we found that the failure to enhance M100 auditory sensory responses to information in the external environment during the listen condition resulted in the M100 listen and speak responses being similar in patients. These findings suggest that patients with SZ have impaired efference copy mechanisms from frontal neural sites to the auditory cortex, resulting in degraded auditory self-predictions about what they expect to hear while speaking. Degenerated efference copy mechanisms to predictable self-generated sounds and impaired auditory responses to less-predictable external sounds resulted in similar M100 responses in patients with SZ during listening and speaking. These failures indicate that patients were unable to generate reliable auditory predictions about the source of external versus internally generated input. Such impaired auditory predictions increased the likelihood of misattributing the source of inner self-generated thoughts/speech as being externally produced, giving rise to disruptions in agency judgments on the high-level RM task and psychotic disruptions in RM that contributed to hallucinations in SZ (2,3,8,9,15,30). These results were not due to differences in latency timing to reach peak M100 responses; they were only due to degraded M100 amplitude auditory sensory responses in the listen condition that were specific to the left hemisphere, which is known to have dominance for language processing. Normally in HCs, left-lateralized M100 and SIS responses are consistent with the typical dominance of the left auditory cortex for speech and language processing. The absence of this lateralization in people with SZ may reflect a disruption in the specialized role of the dominant hemisphere in predictive auditory processing. This is consistent with previous studies showing that auditory verbal hallucinations and language-related abnormalities in SZ were associated with structural and functional deficits in the left STG (36–38). Thus, reduced interhemispheric asymmetry may reflect more generalized dysfunction in auditory-language networks, including altered interhemispheric connectivity or failure to develop typical hemispheric specialization.

Our previous fMRI studies revealed that patients with SZ showed hypoactivation compared with HCs in medial frontal and L.pSTG/S sites associated with impairments in both self-agency judgments and external-agency judgments on the RM task (4,5,39). Here, we replicated and extended these previous fMRI findings using MEG in a different sample of HCs and patients with SZ and provide the first evidence for impairments in agency during a high-level RM task that is linked to degraded A1 M100 auditory sensory activity in the L.pSTG/S during a low-level SM task, thus providing a unitary basis for the underlying mechanisms that contribute to hallucinations in SZ. Previous studies using EEG have shown that medial frontal activity immediately prior to speech onset is strongly correlated with SIS response shown by N1 suppression (during speak minus listen conditions) approximately 100 ms after speech onset (15,40). These data suggest that the brain generates efference copies via medial frontal activity immediately prior to self-generated actions that represent highly reliable self-related predictions that model the expected sensory outcome of self-generated actions during RM and SM tasks underlying agency (5,12,41–43). In HCs, this medial frontal activity prior to vocalization is strongly correlated with subsequent suppression of auditory responses to the spoken sound in the L.pSTG/S (15,40). In contrast, in the current study, we found that patients with SZ showed degraded neural M100 auditory sensory responses to information in the external environment in the L.pSTG/S in the listen condition that drove impaired M100 SIS responses during highly predictable self-generated speech, indicating degraded auditory predictions (2,3,9). Such degraded auditory sensory predictions blurred the boundary between the source of inner self-generated thoughts/speech and external sounds, giving rise to disruptions in agency judgments during RM and hallucinations in SZ.

There are some limitations of the current study. While the current task design does not allow us to examine the origin of the degraded auditory predictions or whether they arise from impairments in generating versus updating internal predictive models, our interpretation is that disruptions in agency are grounded in the predictive coding framework. Previous and ongoing work suggests that impaired SIS responses in SZ may reflect a combination of inefficient sensory gating mechanisms and disrupted frontal-to-temporal cortical communication, particularly impairments in efference copy signaling from frontal to auditory regions (44–46). These disruptions may lead to degraded or imprecise prediction signals during inner thoughts/speech that could be perceived as voices coming from sources other than the self (2,3,16).

We also acknowledge that impairments in both self-agency and external agency may also reflect broader higher-order cognitive deficits (e.g., memory, executive functioning) that could influence agency impairments on the RM task. Thus, agency impairments observed during RM may partially reflect general cognitive dysfunction, in addition to predictive coding deficits. In contrast, the SM task is deliberately designed to minimize such cognitive demands (e.g., attention, memory, visuospatial, or executive functions) and more directly isolates predictive coding processes essential for sensing auditory stimuli. For example, attention is controlled by using identical vowel sounds (/ɑ/) in both speak and listen sessions, matched on acoustic and attention properties, and SIS is defined as the within-subject difference in the MEG M100 auditory sensory response (listen – speak). This subtraction combined with the early 100 ms MEG M100 window minimizes attentional confounds. While we propose a unified predictive coding framework across SM and RM tasks, we recognize that these paradigms engage distinct levels of processing; SM isolates low-level auditory sensory prediction mechanisms during speech, whereas RM requires both low-level prediction coding mechanisms and higher-order cognitive processes for accurate encoding and retrieval of verbal information in the RM task. Thus, although our results suggest shared mechanisms for linking low-level sensory predictive coding mechanisms during speech with higher-order agency impairments during RM and proposes that impairments in agency during RM may stem from foundational disruptions in predictive coding during SM, these findings are correlational in nature. In other words, impaired M100 auditory responses were associated with agency deficits and hallucination severity, but potential mediating factors such as antipsychotic medication dosage, illness duration, and cognitive impairment may also contribute. Consequently, this study alone cannot establish causal links between M100 auditory responses, agency, and hallucination severity. Future neuromodulation studies will be necessary to test causal links as to whether restoring M100 sensory responses that improve predictive coding mechanisms during SM tasks can directly improve agency during RM and reduce hallucinations in SZ.

### Conclusions

We provide a novel perspective for investigating the neural mechanisms underlying agency within 2 distinct SM and RM frameworks and provide a unitary basis for external agency during a high-level RM task that is linked to the fundamental ability to sense and respond to external sounds during a low-level SM task (e.g., shown by enhanced M100 primary auditory sensory responses in the L.pSTG/S). The current findings not only provide innovative functional biomarkers for understanding the underlying neural basis of agency but also provide the first step toward applying precision medicine–guided neuromodulation targets within medial frontal and L.pSTG/S sites to test causal links between predictive coding mechanisms, agency, and hallucinations in SZ (4,5,12,23). Specifically, the current findings suggest that patients with SZ will benefit from directly targeting and enhancing neural activity in the L.pSTG/S that will augment auditory sensory responses to information in the external environment that will drive improved SIS while speaking, thus making it easier for SZ to distinguish the source of inner thoughts/speech from external speech and will potentiate reduced hallucinations. In conclusion, the current research creates a path toward developing new personalized neuromodulation treatment interventions that target medial frontal and L.pSTG/S sites to improve self-agency and external agency, respectively, and that will be particularly useful for patients with psychosis disorders who exhibit severe impairments in both self-agency and external agency for maximally reducing hallucination severity.

## Figures and Tables

**Table 1.** Demographic and Clinical Profiles of HC and SZ Groups

	HC, *n* = 30	SZ, *n* = 30	*p* Value
Age, Years	40 (15)	38 (14)	.60
Gender, Female/Male	10/20	9/21	.78
Race/Ethnic Profile			
Asian	5	3	.45
Black or African American	4	5	.72
Other (more than 1 race)	1	1	..99
White	20	21	.78
SAPS Hallucination Severity	NA	2.6 (1.9)	NA
CPZ Equivalents	NA	254 (240)	NA
Illness Duration, Years	NA	11.6 (10.2)	NA

Values are presented as mean (SD) or *n*.

CPZ, chlorpromazine; HC, healthy control participant; NA, not applicable; SAPS, Scale for the Assessment of Positive Symptoms; SZ, schizophrenia.
